# Social media as a space for support: Young adults' perspectives on producing and consuming user-generated content about diabetes and mental health

**DOI:** 10.1016/j.socscimed.2016.10.006

**Published:** 2016-12

**Authors:** Gillian Fergie, Kate Hunt, Shona Hilton

**Affiliations:** MRC/CSO Social and Public Health Sciences Unit, University of Glasgow, 200 Renfield Street, Glasgow G2 3QB, UK

**Keywords:** UK, E-health, Social media, Diabetes, Mental health, Health experiences, Social support

## Abstract

Social media offer opportunities to both produce and consume content related to health experiences. However, people's social media practices are likely to be influenced by a range of individual, social and environmental factors. The aim of this qualitative study was to explore how engagement with user-generated content can support people with long-term health conditions, and what limits users' adoption of these technologies in the everyday experience of their health condition. Forty semi-structured interviews were conducted with young adults, aged between 18 and 30 years, with experience of diabetes or a common mental health disorder (CMHD). We found that the online activities of these young adults were diverse; they ranged from regular production and consumption (‘prosumption’) of health-related user-generated content to no engagement with such content. Our analysis suggested three main types of users: ‘prosumers’; ‘tacit consumers’ and ‘non-engagers’. A key determinant of participants' engagement with resources related to diabetes and CMHDs in the online environment was their offline experiences of support. Barriers to young adults' participation in online interaction, and sharing of content related to their health experiences, included concerns about compromising their presentation of identity and adherence to conventions about what content is most appropriate for specific social media spaces. Based on our analysis, we suggest that social media do not provide an unproblematic environment for engagement with health content and the generation of supportive networks. Rather, producing and consuming user-generated content is an activity embedded within individuals' specific health experiences and is impacted by offline contexts, as well as their daily engagement with, and expectations, of different social media platforms.

## Highlights

• The online activities of the young adults in this study were diverse.
• Levels of engagement with user-generated content seem related to offline experience.
• We suggest three main ‘types’ of users: prosumers; tacit consumers and non-engagers.
• Some characteristics of users, such as gender, did not relate to ‘type’.
• Identity management acts as a specific barrier to content production about health.

## 1 Introduction

Rapid developments in platforms that facilitate participatory internet activities have broadened users' opportunities for production and consumption of online content (e.g. Kaplan and Haenlein, 2010). Websites such as Facebook, Twitter, YouTube, blogs and wikis, collectively termed social media, facilitate many-to-many communication in contrast to traditional one-to-one personal communication and one-to-many media communication (Hawn, 2009). Since the mid-2000s social media have been widely adopted by users and become embedded features of contemporary life for many (Van Dijck, 2013). While the functionality and popularity of platforms may fluctuate, social media have become well established as sites for the presentation and management of identity (Papacharissi, 2010, Marwick and Boyd, 2011), the organisation of sociable practices (Boyd, 2007), and participation in community-based activity (Burgess and Green, 2009).

Health-related content has been a key feature of users' contributions to the online environment since people started to incorporate accounts of health and illness into personal homepages (Hardey, 2001). Social media allow for more convenient and widespread sharing of images, videos and comments related to health (McNab, 2009) and provide alternative resources for health information-seekers (Fergie et al., 2015). Ziebland and Wyke (2012) have identified seven domains by which people's health is impacted by the sharing of health experiences online (finding information, feeling supported, maintaining relationships with others, affecting behaviour, experiencing health, learning to tell the story, and visualising disease), all of which can be facilitated by various forms of social media. However, the importance of social media for users' health experiences is not always fully recognised. A recent review of policy documents on health and social networking sites suggests growing use of consumer-related marketisation rhetoric but a lack of recognition of the consistent role of social networking sites in facilitating peer-support and information sharing amongst people managing long-term health conditions (Hunt et al., 2015). Indeed, given the propensity for social media use amongst those experiencing chronic health issues (Fox and Jones, 2011), this group provides a key focus for developing a more nuanced understanding of the adoption and adaption of social media technology for the generation and exchange of health information.

‘Users’ have been variously characterised according to offline characteristics, such as social position and age. Some authors have suggested that online technologies exacerbate existing social inequalities, creating a ‘digital divide’ (Norris, 2001). Others have characterised young people as ‘digital natives’, a generation of internet users accustomed to the online environment (Tapscott, 1998, Prensky, 2001). Alternatively, Dutton et al. (2013) suggest five groupings of ‘users’ based on their perceptions of what the internet can provide for them, ranging from “enjoyable escape” to “problems” (p. 6). Nettleton et al.’s (2004) study presents a nuanced account of users' engagement with online health resources. The typology they suggest is based on relationship with the internet (problematic, episodic or domesticated) and form of reflexive engagement (instrumental or affective). This typology provides a useful starting point for understanding users' online practices. However, exploring people's practices in relation to user-generated health content specifically could offer new insights around contemporary internet use and engagement with health information.

In a recent UK survey of internet users, 25% of respondents reported accessing or sharing user-generated health-related content online. A minority of super-users (7.5%) acted as prolific contributors of user-generated content for others' consumption (O'Neill et al., 2014). The authors suggest two areas for further research: the activities of those who do not actively contribute health content but who consume content as “lurkers”; and the characteristics and requirements of a minority of internet users who reported no awareness of health-related user-generated content. To date, little research has explored the perspectives of these groups, or those characterised as “super-users” or “prosumers”, i.e. users who are engaged in the simultaneous processes of production and consumption (Kaplan and Haenlein, 2010, Ritzer and Jurgenson, 2010).

This study builds on previous characterisations of ‘users’ to explore engagement with health-related user-generated content in young adults with experience of long-term health conditions. Given the importance of exploring health-related social media use amongst those with long-term health issues (Fox and Jones, 2011, Hunt et al., 2015), we elected to focus on diabetes and common mental health disorders (CMHDs), such as depression, anxiety, panic and post-traumatic stress disorder (Nice, 2011), as exemplars. Entwistle et al., (2011) suggest that exploring experiences across a sample of participants with different health issues can enrich analyses because of the different information-seeking and decision-making challenges each issue presents. Diabetes and CMHDs involve different considerations for people in relation to: diagnosis, treatment options, information needs and decision-making. However, both involve some degree of self-management (Sterling et al., 2010). Moreover, both health issues have precipitated the development of high levels of user-generated content and vibrant online communities (Yonker et al., 2014, Hilliard et al., 2015).

Current research does not provide a detailed account of the ways that different social media platforms are experienced by people, particularly young people who increasingly use social media for social purposes and to engage with health content. Understanding current online practices is important for informing development of online health resources to support people's experiences of both diabetes and CMHDs. Current research also lacks exploration of the context of people's engagement with user-generated content and the barriers to contributing such content. This research was designed to explore young adults' experiences of consuming *and* producing health-related user-generated content as featured on popular social media sites, in the context of their day-to-day management of their health conditions. Our aim is to gain new insights into how people with long-term health conditions engage with user-generated content, and what barriers, if any, limit users' adoption of these technologies for supporting them in their health experiences.

## 2 Methods

Following a broadly interpretivist approach (Green and Thorogood, 2013), we used qualitative interviews to explore young adults' perspectives; similar methods have been used by others who have explored young people's social media use (Das, 2011). Ethical approval was gained from the University of Glasgow, College of Social Sciences Research Ethics Committee.

### 2.1 Sample

A purposive sample of men and women, aged 18–30 years, with experience of either diabetes or a CMHD, was selected for interview. Young adults were of particular interest to this study because of their life-stage and their potentially rich online experience. The life-stage of young, or ‘emerging’, adulthood has been identified as an important period of transition (Arnett, 2000), and ‘emerging’ adults as a group with unmet health needs (Marshall, 2011). Young adults (16–24 years) in the UK are also more likely to use the internet for social networking than all other internet users (83% of young adults visit social networking sites more than once a day) (Ofcom, 2014). Furthermore, in relation to the use of online support groups, a review of research on online communities for supporting experience of depression suggests most users were in their mid-20s to mid-40s (Griffiths et al., 2009). By identifying young adults as the population of interest, the focus of the study was narrowed to individuals who might share a common awareness of social media technologies.

Forty semi-structured interviews were conducted by GF between November 2012 and May 2013. Table 1 contains information on participant characteristics. The sample was designed to include similar numbers of men and women and similar numbers of people with experience of diabetes and CMHDs to allow exploration of differences and similarities between these sub-groups.

Participants were recruited both offline and online (see Table 1). In order to specifically target young adults, information about the study was displayed in further/continuing education institutions (offering basic skills training and vocational qualifications) and higher education institutions (offering degree level qualifications). Gatekeepers working in organisations which support young adults with diabetes or CMHDs also distributed study information to potential participants, and some participants recommended the study to their peers. Study information was also posted online in Facebook groups and forums for discussion of diabetes or CMHDs.

### 2.2 Data collection

Interview locations included cafes (n = 17), participants' homes (n = 12) and university buildings (n = 11), based on participants' preferences. After discussing the aim of the study and the processes for safeguarding data and participants' identities, participants provided written informed consent.

The first half of the interview focused on participants' experiences of their particular health issue. They were asked open questions about first experiences/diagnosis, how they had learned about their health issue and what supported their management of it. In general, participants gave detailed accounts of their experience, often foregrounding the provision or lack of sources of support. Next, participants were provided with a tablet computer to consider a selection of online content related to their condition and asked to discuss their perceptions of this content and their experiences of engaging with similar content. The examples of content included Facebook, YouTube and Twitter pages, featuring different types of online content (images, text extracts and videos). Examples were selected to highlight popular forms of user-generated content relevant to either diabetes or CMHDs, and included original user-generated content and responses from other users (ratings and comments). Participants discussed their perceptions of this content and their experiences of engaging with similar content. The content served as a stimulus for wider discussion of perceptions and practices related to different platforms and content, rather than as the sole focus for structured interview questions. Participants were encouraged to explore other sites and use the tablet to illustrate types of content they preferred or disliked based on previous experience.

Interviews lasted between 40 and 90 min and were digitally recorded with participants' permission, transcribed verbatim and anonymised. After forty interviews all authors reviewed the transcripts and concluded that a rich volume of data had been collected which allowed for identification of key themes.

### 2.3 Data analysis

The first stage of thematic analysis, conducted by GF, was based on principles of Grounded Theory, including inductive coding, constructing categories and continuously comparing codes (Floersch et al., 2010). Codes and categories were grouped into wider themes to produce thematic networks (Attride-Stirling, 2001). These were discussed by all authors, on the basis of their independent reading and interpretation of the data, and refinements were agreed. Throughout the analysis accounts were compared systematically for differences and similarities between men and women, and between experiences of diabetes and CMHDs. A matrix was constructed based on participants' descriptions of offline support and engagement with health-related user-generated content to produce a typology.

## 3 Engagement with health-related user-generated content in relation to offline support

Almost all participants described health-related content as an inevitable feature of social media but their levels of engagement varied. Our analysis suggests that engagement with health-related user-generated content is related to context and environment, in particular experiences of offline support. Based on analysis of these two dimensions, engagement with user-generated content and experiences of offline support, we suggest a typology of three main ‘types’ of users. We refer to those who described greatest offline support and engaged less regularly with user-generated content as ‘non-engagers’. Those who described least support in their offline networks and engaged most frequently in production and consumption of health-related user-generated content are referred to as ‘prosumers’. Those who described some degree of offline support and consumption of health-related content, but not production, are referred to as ‘tacit consumers’. Table 2 summarises some of the key characteristics of these types.

### 3.1 ‘Non-engagers’

‘Non-engagers’ described well-developed offline support and limited engagement with health-related user-generated content. Alongside discussing how they avoided accounts of other people's experiences online, several talked about their reliance on close relatives for support in managing their illness, including some with first-hand experience of their condition. Andy, who was diagnosed with Type 1 diabetes as a child, reflected on engagement with user-generated content about diabetes:

I think people might be more drawn to using these kind of things if they don't have somebody like I did with Mum and Dad, who sort of really cared for the whole thing, got as much information as they could […] other people might have different family backgrounds, […] they might have to look for it themselves, and that's gonna be difficult for them, but I mean having read the guys' [comments] and people they can talk to [the online community] who actually know what they're talking about, [it is] quite comforting for them to have so they probably use that facility quite a lot, eh I know I would if I didn't have the support of Mum and Dad (Andy, diabetes, 22).

Andy frames his description of parental support in contrast to the support experienced by people who use online resources. Similar remarks were made by other participants who encountered a high level of offline support for CMHDs. Peter commented:

I didn't really look it up [online], […] after going to see my GP and then [mental health professional], I didn't really look at any other ways because [she] helped a lot […] whereas if it was getting worse and [she] wasn't helping, I would have […]. Whereas if it [had not] helped, then I'd still be looking for that, for that open door to kind of make me feel better (Peter, CMHD, 22).

Peter articulated why, for him, finding the support of a mental health professional who “helped a lot” meant that looking elsewhere, including online, was not necessary. However, he recognised that this might not have been the case had circumstances been different.

For ‘non-engagers’, consistent, specific and reliable offline support seemed to preclude the need for engagement with health-related user-generated content online. Instead, close relatives with first-hand experience, parents who had developed expertise, or valued health professionals acted as adequate sources of information and support. Perhaps then, young adults who have established these supportive resources offline may see little need to explore others' experiences through social media and are less likely to identify with people who produce user-generated content. While this is not problematic, it does pose a challenge for producers of online peer support resources, since ‘non-engagers’ are unlikely to be at ease with consuming or producing health-related content.

### 3.2 ‘Prosumers’

Unlike ‘non-engagers’ who described reliable, instrumental and emotional support from family, friends or formal services, ‘prosumers’ described a range of experiences of low levels of offline support, and high levels of production and consumption of user-generated content. In some cases, this reflected being diagnosed or first experiencing their health issue as a teenager or young adult and taking sole responsibility for managing their health issue from the outset rather than relying on parental support. For instance Poppy commented on her experience of being diagnosed with diabetes at the age of 26:

The human side and the actual day to day mechanics of treating it [diabetes] was a lot harder and it only dawned on me it was much harder sort of a few months in [after being diagnosed].

Poppy went on to discuss accessing supportive user-generated content online whilst she was pregnant:

Eventually I found the support group on the internet but that was more an emotional thing than finding information. […] I'm actually a member of a Facebook group […] I'm very active on there at the moment. […] There's conversations going on at the moment about HbA1c (Poppy, diabetes, 30).

Poppy described how she initially expected that her professional expertise, as a vet, would be adequate self-management practices. However, when she found this was not the case, she searched for additional peer support online. Similarly, Eleanor began both producing and consuming content online when she experienced an absence of emotional and instrumental support elsewhere. She described how, when she first experienced depression at 18 years old, she lacked support from her family:

There's so much stigma and I think a lot of people are still quite scared, I mean I know when I first got diagnosed my Mum and that didn't really understand it, […] you know, ‘You're just a nervous wreck’ or whatever, it took them so long to kinda realise it was something proper.

Eleanor went on to discuss her independent exploration of a range of online content, including user-generated:

I mean you go to the doctor for your basics but the main source of information is online I think because I depend on, you know, support groups on Facebook or, you know, [named mental health forum], things like that because that's where people come. […] I mean the professionals do do their best to help you where they can but even they're referring you to things online now, […] my family try and say things, but they've not really been through it, […] I'd rather find things out on my own (Eleanor, CMHD, 26).

Eleanor discussed the many ways that she engaged online with others with experience of mental health problems, to go beyond the “basics” provided by health professionals and the input of people without experience. Similarly, early in his account, Anthony discussed his conception of family and professional support:

To be honest, I tried to keep [my parents] out of it as much as possible because it was already very clear straight from the off that this was going to be about me. […] Obviously [health professionals] spoke to my parents about diet and well, […] in retrospect, the advice I think they gave my parents was incorrect but that's kind of another issue […] there's an insistence that diet needs to be based around 60–70% starchy carbohydrates, whereas, and this is actually from using the internet and communicating with other people, I've learned that actually most people have a very different experience, in fact that's actually one of the worst possible diets (Anthony, diabetes, 28).

For Anthony, diagnosis at age 14 and the adoption of an independent approach to self-management, seem to have prompted him to develop as a ‘prosumer’ of diabetes-related content, rejecting parental support and prioritising online communication.

Unlike ‘non-engagers’, ‘prosumers’ described inadequate sources of offline support or a desire to approach their health issues independently and, as a result, they seemed drawn to engage with user-generated content. Their role as health content ‘prosumers’ was linked to external factors such as illness trajectory, with their age at diagnosis and current health experiences leading them to develop ‘prosumption’ practices online as a means of establishing supportive resources. Social media, therefore, seem a particularly important resource for young adults without adequate sources of offline support.

### 3.3 ‘Tacit consumers’

‘Tacit consumers’ shared a propensity for consuming user-generated content about their health issue without contributing any. They described experiencing some offline support from family, friends or local services, but exploring user-generated content as a means of supplementing these resources. For instance, while browsing Facebook pages related to depression and anxiety, Leah commented:

I mean, I always knew that I wasn't alone, but when you're right down there, you do feel as if 'this is it'. […] I felt so low […] you know what it's like, when you flick through your news feed or your Twitter and you see something like the wee pictures that I kept seeing […] it kind o' pulls you back (Leah, CMHD, 26).

Unlike the ‘prosumers’, Leah did not use social media to engage directly with groups of users to discuss her experience of CMHDs. Her engagement with user-generated content on social media sites seemed to consist largely of consuming visual content featuring inspirational quotes shared by other Facebook users. Leah related this engagement to counteracting feelings of loneliness and isolation.

Similarly, Mhairi described her early experiences of managing diabetes in the context of her day-to-day life as characterised by anxiety. She discussed how user-generated content had allayed these anxieties:

I don't know if you've heard of [website] before? It's fab, it's a blogger […], she's got a Twitter site and she also writes blogs, […] I think she's about 25 or something. But, she writes about diabetes but in terms of her life as well, and it's all kinda—it's quite positive the way she puts a slant on it […] So, it's kinda that peer-support, kinda thing […] I think I would post on it if I knew that what I was saying was definitely a hundred percent accurate. Or, if it was about maybe my experience. But, no, I've never posted anything (Mhairi, diabetes, 28).

Mhairi identified a specific blogger, of a similar age, as a particularly useful source of content across different social media platforms. She associated consumption of this content with peer support. Mhairi's comments, however, also suggest she is not currently prepared to contribute content. Such comments were typical of the ‘tacit consumers’. Paul (aged 30), who experienced depression and low mood related to a gambling addiction, talked about his perceptions of contributing content: “I know it would probably be useful to actually like speak to other kinda like-minded people on something like that [Facebook page about depression] but it isn't something that I've actually ever done”. Unlike ‘prosumers’, ‘tacit consumers’ did not seem at ease with producing content, despite drawing on others' accounts of diabetes or CMHDs. Some barriers to contributing health-related content are discussed in more detail.

### 3.4 Temporality and movement between ‘types’

Most participants fitted broadly within the three types described. However, a few accounts resonated with both ‘non-engagers’ and ‘tacit consumers’, but did not conform completely to the characteristics of either group. Generally, these participants described being well-supported and rarely consuming user-generated content, yet described engaging with some forms of user-generated content in specific circumstances. Penny, who rarely consumed user-generated content, discussed accessing content at a time when she lacked alternative sources of support.

I didn't really have many friends, cos it's when we lived in England and [partner] was away and spoke to my Mum and that but it was good to see other people speaking about it [online] and what their thoughts and that were on it (Penny, diabetes, 27).

The temporary absence of Penny's partner seems to have prompted her to access advice in a way which she suggested was unusual for her. Furthermore, two participants' experiences were not aligned with any of the types described, perhaps because of the complexity of their health conditions; in both cases, the participants had not yet been given a specific or satisfactory diagnosis. These instances illustrate the limitations of conceptualising all users as three distinct groups. Whilst the typology summarises accounts of online practice and experiences of offline support, it does not reflect the dynamic nature of either the online environment or people's lives and health experiences. Unforeseen changes in health status, self-management practices or availability of supportive resources are likely to affect, or disrupt, online practices.

## 4 Users' considerations of identity and audience on social media sites

Despite the range of ways in which the three ‘types’ of participants engaged with health-related user-generated content online, all discussed how considerations of their identity and presumed audience of social media impacted their online practices.

### 4.1 Producing health-related content

During the interviews many participants reflected on the undesirability of contributing any health-related content to Facebook, since this platform was seen primarily as a space for the conscious construction of a positive identity. As such, the inclusion of references to diabetes or mental health could jeopardise this. These concerns were articulated in particular by ‘non-engagers’ and ‘tacit consumers’. Fraser discussed his concerns:

Facebook, links into your sort of general, your wider, you know, Facebook profile, and it's [mental health issue] not something you would necessarily want to be public. And if someone's stalking you on Facebook, not stalking, but, you know, someone is looking at your Facebook page and they see that, that's not necessarily something you'll want them to see (Fraser, CMHD, 28).

Fraser's comments suggest the active decisions he makes about what personal content he presents to those viewing his profile. Alluding to health issues on Facebook was described as “too public” (Alistair, CMHD, 21), “cringey” (Liz, CMHD, 19) or like “shouting it from the rooftops” (Leanne, diabetes, 23). Some elaborated on their reluctance to post health-related content, relating their rationale directly to concerns about identity:

People would think “She's moaning on about it’” [diabetes] and I don't want to be a moaner, […] don't want to be just known as the girl who has diabetes and is always going hypo and things (Penny, diabetes, 27).

Many participants, including a number of ‘prosumers’, expressed concerns about being perceived as “the girl/boy who has diabetes/mental health issues” or as someone who was always “moaning on” about their illness.

Similarly, when asked about ‘liking’ a mental health charity Facebook page, Simon said:

People can see what you ‘like’, so, yeah that's strange isn't it ‘cause I would actually, I would like to ‘like’ that page, but […] It's strange ‘cause you feel like “oh”, I'd feel a bit vulnerable […] it seems more like, Facebook, you just, you kinda promote yourself or, yeah, you promote a version of yourself, that you maybe think people would like to … like to ‘like’ [laughs] (Simon, CMHD, 30).

For many participants, ‘liking’ a page was a statement of endorsement and personal association with the subject of that page. For some, like Simon, health charity or support group pages were not deemed suitable for ‘liking’ since they might detract from the presumed purpose of Facebook, to promote the ‘right’ version of yourself.

Although most avoided posting health-related status updates, two participants discussed using Facebook to share health experiences. When asked if she ever posted about her diabetes on Facebook, Ingrid responded:

I've got my manager on my Facebook as well so I'm very aware of her knowing that I'm not just faking it and taking the piss and stuff so um, I do post how sick I'm feeling or […] I try and like make jokes about it and stuff just so that people are aware and don't just like think I'm being grumpy or something for no reason (Ingrid, diabetes, 23)

Unlike the other participants, Ingrid discussed using Facebook to present her health status to her Facebook ‘friends’. By sharing her experiences on Facebook she hoped to both justify her sickness absence to her manager, and provide a rationale for her mood to friends.

Joe also used Facebook to contribute health-related content, acknowledging the potential undesirability of using the site in this way:

You know, I will quite often just sort of tell Facebook that I'm feeling really anxious or I'm feeling really down or I'm just confused and things like that. I try to avoid what they call ‘vague booking’, which is, you know, posting something really vague-sounding so that people'll ask you a question about it (Joe, CMHD, 28).

While Facebook is often conceived as a site for discussion of the mundane, details of health experiences were not always deemed suitable content for status updates due to the potentially negative impact this could have on individuals' presentations of themselves. The small minority who did use Facebook ‘status updates’ to share experiences of their health issues justified the practice by providing either a practical rationale or suggesting boundaries around how posts are phrased.

Making active decisions about what characteristics to include or privilege whilst presenting ‘a version’ of themselves online seems to be an over-riding concern when engaging with health-related content on social media, regardless of health issue or level of ‘prosumption’. The production of content on Facebook, in particular, seemed restricted by considerations around the presentation of identity to the ‘friends’ they conceived as their ‘audience’. This concerns extended to online interactions, such as ‘liking’ Facebook content related to health conditions. For a small number of participants, however, producing health-related content served to integrate carefully-considered articulations of health experiences into their online self-presentation.

### 4.2 Expectations of appropriate online practices in social media spaces

Participants' conceptions of the purpose and remit of social media spaces, and related audiences, were crucial determinants of how they interacted with health content on social media sites. Many conceptualised Facebook as a social space and not a space for ‘serious’ or personal health-related discussion. Alistair, who had experienced depression and anxiety periodically since childhood, commented:

Putting stuff that's personal on the internet, to me, is weird, […] it's just something that I wouldn't do […] like there's a guy who, he lost his mum […] and he posts about it quite a lot, like on her birthday, […] I don't think Facebook's the place for anything like, for serious discussion, for things as important as that (Alistair, CMHD, 21).

Alistair's account echoes other participants' concerns that health-related content is too ‘serious’ or ‘personal’ for Facebook. Other social media channels were seen less as wholly social spaces and more appropriate for serious discussion. Although less commonly discussed by participants, Twitter, in contrast to Facebook, was sometimes presented as a more appropriate site for health-related debate:

Facebook's not really a place you have discussions, people will post something and say “great comment” and that's kind of the gist of it […] [Twitter] has a little bit more anonymity for me because, generally, the people who are following on Twitter are not actually personal friends. […] They do actually have kind of debates on Twitter, I've actually tried to engage [diabetes charity] a few times before. […] What they were doing, which is really effective, is they were hashtagging everything so there was like a whole sort of stream of people posting on the same tag […]. I also asked a few questions about diet as well, [and] at the end of that week I had another like 30 or 40 followers (Anthony, diabetes, 28).

As a ‘prosumer’ of online content focused on developing a critical understanding of many aspects of diabetes, Anthony deemed Twitter a more appropriate space to challenge medical discourse and orthodoxy than Facebook.

Both consumption and production of health-related user-generated content seem to be impacted by participants' expectations of particular online spaces and their daily engagement with different social media platforms. Their conceptions of the purpose and remit of these spaces, and how other users interact within them, seem to be crucial determinants of ‘appropriate’ content production. Therefore, for many young adults, even the most active ‘prosumers’, the social and environmental aspects of the online context impact engagement with user-generated content.

## 5 Discussion

Increasing levels of “prosumption” (Kaplan and Haenlein, 2010) have meant that the volume of health-related user-generated content online has increased exponentially. Our study provides new insights into the range of ways young adults with chronic health conditions engage with user-generated content, the offline context of this engagement and the barriers to using social media for health purposes. The online activities of the young adults who participated in this study were diverse, our analysis suggested three main types of users: ‘prosumers’; ‘tacit consumers’ and ‘non-engagers’. Similar to Nettleton et al.’s (2004) typology, which categorised general internet use for health purposes, these types reflect the role of user-generated content as a resource in people's everyday lives, embedded within their social context and current health-related experience. Indeed, the typology we propose explicitly references how engagement with sources of support offline might impact engagement with user-generated content as a means of accessing accounts of other people's experiences and peer support.

The ‘prosumers’ and ‘tacit consumers’ we identify are aligned, in part, with quantitative findings around health-related social media use, which suggests two key user-groups are ‘super-users’ and ‘lurkers’ (O'Neill et al., 2014). Our research suggests some of the circumstances which might lead to users occupying these types, in particular their offline experiences of support and related factors such as age at diagnosis and current phase of illness. Other characteristics of users, such as gender, did not relate to ‘type’. Some have reported differences in online information seeking by gender, with women being more likely to use the internet as a resource for health information (Ybarra and Suman, 2006, Powell et al., 2011). A systematic comparison of the accounts of men and women, revealed no notable differences in engagement with user-generated content. Gender differences in help-seeking behaviours, particularly in relation to mental health are often presumed to be important, although recent research suggests that they may not be as marked as previously assumed (Wang et al., 2013). Both male and female participants identified opportunities for supportive resources to be established through social media and cited concerns about privacy as limiting their engagement, with little indication of different needs or expectations according to gender. Furthermore, few differences emerged across the two health issues, despite the inherent differences in information seeking and decision-making challenges across different health issues (Entwistle et al., 2011). Participants with both diabetes and CMHDs were represented within each ‘type’, and although there were some differences in how their health issues impacted online engagement, there were many similarities related to issues of support and engagement preferences. More broadly our findings confirm the importance of peer support as a key means of supporting long-term health issues and self–management (Gallant, 2003, Embuldeniya et al., 2013). They also confirm the potential of the internet in offering opportunities to access other people's experiences in a way that can positively impact users' own experiences (Ziebland and Wyke, 2012), especially where offline peer-support is lacking or deemed inappropriate.

Alongside being characterised in the research literature, ‘users’ are also constructed by producers of online resources (Oudshoorn and Somers, 2006). Therefore, when developing online resources to support users' health experiences, it is important to understand the variation in young adults' engagement with user-generated content. Profiling users according to the adequacy of their *offline* support may be a useful means of understanding their *online* practices. In particular, those who describe having little offline support may be more likely to become active ‘prosumers’ of content. Producers of online resources should therefore attempt to facilitate the close peer-support, experience-sharing and detailed technical discussion that these users may lack. However, relationships between offline support and online engagement could continue to change over time, as both online technologies and users' practices or circumstances change, requiring continued innovation of online resources.

For those attempting to use social media in health interventions, understanding common reservations about engaging online, as described here, seems crucial. While the activities of consumption and production of content through sites like Facebook, Twitter and YouTube were very familiar to these young adults, their accounts conveyed complex decision-making processes, particularly around content production, embedded in everyday online practices. In particular, the importance of day-to-day identity management online seems to act as a specific barrier to content production.

In accordance with previous research (e.g. Boyd, 2007; Ellison et al., 2006), Facebook was identified as an important space for constructing personal and group identities. The participants' accounts seem aligned with social-interactionist conceptions of identity (Mead, 1934, Goffman, 1959), with users of social media sending signals to establish their identities through interaction with other users. Posting on Facebook, Tweeting or ‘liking’ content can all be understood as means of performing identities in Goffman (1959) terms, in a way that mirrors offline social interaction. Robinson (2007) has highlighted the online environment as a site for identity performance, asserting that postmodern notions of identity (which suggest the re-creation and re-invention of the self) are less relevant to contemporary online practices:

[…] postmodern accounts of cyberselfing do not prove convincing for today's internet users in light of changing trends in the internet user population and its online activities. […] Like offline self-ing, cyberself-ing is rooted in interaction as understood by Mead; the ‘I’, the ‘me’ and the ‘generalised other’ inform each other as the core of the self-ing project (p. 107).

Our study provides further empirical evidence for Robinson (2007) assertion, particularly in the context of discussions of health experience. The social interactive nature of Facebook and Twitter makes these platforms key sites for identity construction closely related to offline identities and not re-invented selves. While for some users some platforms provide an opportunity for the production of identities linked to their health experience as experts or advocates, many choose not to disclose health experiences on social media profiles in a bid to limit negative implications for identity construction. Although some studies point to the use of social media for the communication of key health messages to young people (Ralph, 2011), and young men in particular (Robinson and Robertson, 2010), it seems important to understand how this context impacts intended users. Explicit concerns about how presumed audiences might view references to health issues on social media profiles, particularly Facebook, may mean that even the seemingly innocuous act of ‘liking’ pages (which initiates subscribtion to content on an ongoing basis) is subject to considerations about identity-management.

Site-specific preferences, such as participants' conceptualisation of Twitter as a more appropriate site for health-related discussion than Facebook, highlights a further tension inherent in using social media to support young adults with health issues such as diabetes and CMHDs. Libreri and Graffigna (2012) commented that few studies explore the differences in the type and tone of content contributed across different social media platforms. Our findings confirm the importance of considering these differences from the users' perspective. Similarly, the lack of emphasis in policy documents on the role of social media for self-management and peer-support (Hunt et al., 2015) suggests more work is needed to understand how best to harness users' enthusiasm for health-related social media use. Our findings suggest that resources should be developed which are in accordance with established online practices, since these shape young adults' uptake of opportunities to engage with health content online. People's everyday engagement with social media, the types of content they prefer and their perceptions of the purpose, potential and cultures of particular online spaces are likely to shape how they will engage with health-related content. In this study, even those who “prosumed” content were careful to adhere to established conventions around what was considered appropriate across different social media platforms. Participants were critical of those who flouted these conventions. Hence, as health departments and charities attempt to utilise social media platforms to reach specific audiences, it seems important to consider each platform separately. In particular, the genres of content a particular platform hosts, and the established conventions related to the platform's interactive features, are key concerns of users as they consume and/or produce health-related content.

Strengths of this study include: the generation of rich qualitative data with a large purposively-selected sample of young adults; and the use of online resources within interviews to prompt reflection on social media environments and forms of user-generated content. However, the study has some limitations. The sample mainly consisted of young adults who were employed or in full-time education and, although online access was not a pre-requisite for inclusion, all participants accessed online technologies daily – many through smartphones. The sample was also limited in other ways (e.g. in relation to ethnicity and socio-economic status). The findings may therefore not be generalisable to other groups, including those with different cultural expectations of health services. A further inevitable limitation relates to the dynamic nature of online practices. Participants' reflections on specific social media sites and online practices were grounded in their day-to-day experiences. Such experiences are subject to rapid changes in preferences and technologies, especially as existing social media sites are supplanted by other platforms.

In summary, this study highlights the complexities of users' engagement with user-generated content for support in their experience of long-term health conditions, in relation to diabetes and CMHDs at least. The findings highlight the range of considerations which influence production and consumption of health content via social media, particularly around identity management and integrating health content into everyday online practice. They also demonstrate how online user engagement articulate with offline sources of support.

## Figures and Tables

**Table 1:** Characteristics of participants.

Gender	Health issue	Pseudonym	Age	Highest level of education	Time since diagnosis or initial experience of health issue (years)	Recruitment (online/offline)
Male	Diabetes	Andy	22	Secondary	>10	Offline
		Anthony	28	Tertiary	>10	Online
		Blake	27	Secondary	>10	Offline
		Byron	18	Tertiary	6	Online
		David	29	Secondary	>10	Online
		Leon	22	Tertiary	2	Offline
		Max	29	Tertiary	>10	Online
		Ronan	28	Tertiary	>10	Offline
		Rory	30	Tertiary	>10	Offline
		Tommy	28	Secondary	>10	Offline
	CMHD	Alistair	21	Tertiary	5	Offline
		Daniel*	25	Secondary	1	Offline
		Euan	28	Tertiary	5	Offline
		Fraser	28	Tertiary	>10	Offline
		Joe	28	Tertiary	>10	Online
		Josh	27	Secondary	2	Offline
		Mike	30	Tertiary	5	Online
		Paul	30	Tertiary	9	Online
		Peter	19	Secondary	1	Offline
		Simon	30	Tertiary	1	Offline
Female	Diabetes	Bronwyn	28	Tertiary	>10	Offline
		Fiona*	22	Tertiary	2	Offline
		Freya	19	Tertiary	9	Offline
		Ingrid	23	Tertiary	2	Offline
		Jill	24	Tertiary	>10	Offline
		Leanne	23	Tertiary	2	Offline
		Mhairi	28	Tertiary	1	Online
		Nicola*	28	Tertiary	>10	Offline
		Penny	27	Tertiary	>10	Offline
		Poppy	30	Tertiary	4	Online
	CMHD	Debbie	30	Tertiary	>10	Offline
		Eleanor	26	Tertiary	8	Online
		Fran	25	Tertiary	8	Offline
		Leah	26	Tertiary	2	Offline
		Liz	19	Secondary	5	Online
		Mia	20	Secondary	6	Offline
		Sarah*	22	Tertiary	7	Offline
		Simone	25	Tertiary	>10	Offline
		Sylvia	26	Tertiary	>10	Offline
		Tara	21	Tertiary	8	Offline

* denotes pilot participants.

**Table 2:**
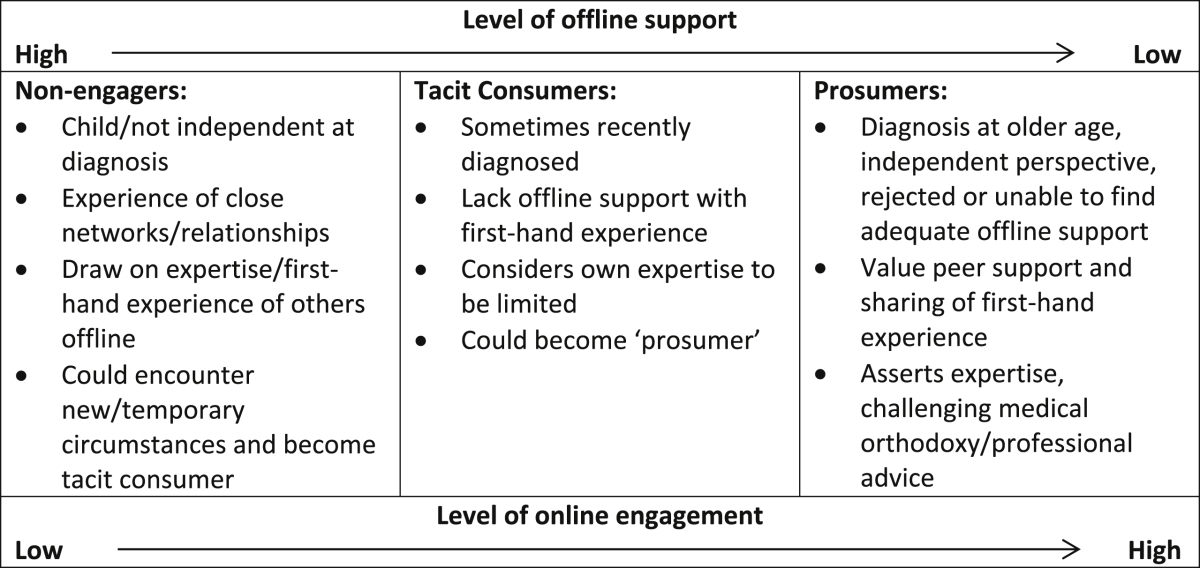
Typology of engagement with user-generated content.
